# Utility of a novel sheath designed for mapping biopsy for preoperative malignant hilar biliary obstruction

**DOI:** 10.1055/a-2787-1325

**Published:** 2026-01-30

**Authors:** Hiroki Sakamoto, Hirotoshi Ishiwatari, Masahiro Yamamura, Takuya Doi, Junya Sato, Yuko Kakuda, Tomoko Norose, Nobuyuki Oike, Teiichi Sugiura, Katsuhiko Uesaka

**Affiliations:** 1 38471 Division of Endoscopy, Shizuoka Cancer Center, Nagaizumi, Japan; 2 38471 Division of Pathology, Shizuoka Cancer Center, Nagaizumi, Japan; 3 12927 Department of Pathology, St Marianna University School of Medicine, Kawasaki, Japan; 4 38471 Division of Hepato-Biliary-Pancreatic Surgery, Shizuoka Cancer Center, Nagaizumi, Japan

**Keywords:** Pancreatobiliary (ERCP/PTCD), Tissue diagnosis, Strictures, Diagnostic ERC

## Abstract

**Background and study aims:**

Mapping biopsy (MB) aids in diagnosing superficial mucosal spread of biliary tract cancer. However, conventional MB is technically challenging, which can reduce the diagnostic yield. This study aimed to assess the usefulness of MB using the newly developed Endosheather (ES) (Piolax, Tokyo, Japan).

**Patients and methods:**

We retrospectively analyzed data from patients who had biliary tract cancer with hilar biliary obstruction and underwent MB. Outcome measures included the overall technical success rate, quality of specimens, impact of MB results on the treatment strategy, and adverse events (AEs), which were compared between the conventional (Conv) group and the ES group.

**Results:**

A total of 91 patients were included. The overall technical success rate was significantly higher in the ES group (92%, 43/47) than in the Conv group (59%, 26/44) ( *P* < 0.05). The rate of samples containing both biliary epithelium and stromal tissue was significantly higher in the ES group than in the Conv group (77% [67/87] vs. 47% [32/68]; *P* < 0.05). MB results affected the treatment strategy in one patient (2.3%) in the Conv group and five patients (11%) in the ES group ( *P* = 0.11). There was no significant difference between the two groups in terms of AEs.

**Conclusions:**

MB using ES significantly improved technical success and specimen quality, providing valuable information for preoperative assessment of patients with biliary tract cancer.

## Introduction

Representative malignant diseases that cause hilar biliary obstruction include biliary tract cancers, such as cholangiocarcinoma and gallbladder carcinoma. The prognosis for these cancers is poor, and radical surgical resection is the only curative treatment 1 2 3 . There are differences in its outcomes between Western and Eastern countries, with excellent results particularly noted from high-volume centers in Japan (mortality rate, 0–1.4%) 4 5 6 7 . These outcomes may stem not only from surgeon skill but also from careful preoperative patient management, including preoperative biliary drainage, liver function tests, and assessment of cancer spread 4 .

To determine the indication for surgical treatment and to select an appropriate surgical method, it is crucial to assess the extent of tumor spread based on preoperative examinations. Imaging tests, including multi-detector computed tomography (CT), magnetic resonance imaging, and endoscopic retrograde cholangiopancreatography (ERCP), are useful for diagnosing the horizontal spread of biliary tract cancer 8 9 10 . However, biliary tract cancer can sometimes spread superficially along the bile duct, making accurate diagnosis challenging.

Mapping biopsy (MB) has been used as a supportive method to diagnose superficial mucosal spread, and the additional information it provides can be beneficial for optimizing treatment strategies. In conventional (Conv) MB, the biopsy forceps are inserted directly into the bile duct from the major duodenal papilla. To obtain specimens above the biliary obstruction, it is necessary to pass the biopsy forceps through the biliary obstruction, which can lead to false positives owing to tumor cell contamination. Furthermore, this method can be technically demanding because inserting forceps beyond the biliary obstruction may result in failure. To address these issues, a biopsy method using a stent delivery system has been introduced 11 12 . However, this method requires adjustment by endoscopists, and it may break during the procedure because of insufficient stiffness. In addition, peroral cholangioscopy (POCS) can be used for MB, but it is also challenging to insert beyond the biliary obstruction and high medical costs hamper its use for diagnostic purposes 13 14 . Under these circumstances, the Endosheather (ES) (Piolax, Tokyo, Japan) has been developed to facilitate forceps biopsy during ERCP. Forceps biopsy using ES yields high sensitivity and accuracy for diagnosing biliary obstruction 15 16 . Nevertheless, to our knowledge, no study has evaluated the utility of MB using ES for biliary tract cancer. Thus, this study aimed to assess usefulness of MB using ES for biliary tract cancer with hilar biliary obstruction in comparison with Conv MB.

## Patients and methods

### Study design

**Fig. 1:**
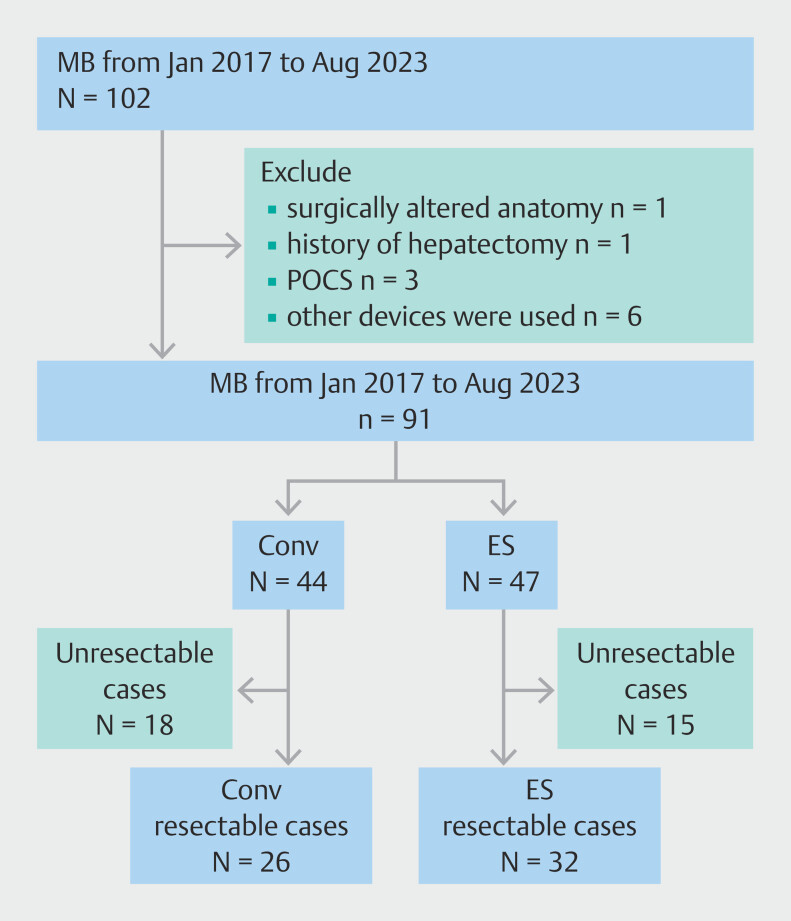
Flow chart of patients in this study. A total of 102 patients underwent mapping biopsy (MB) during the study period. Eleven patients were excluded: one with surgically altered anatomy, one with a history of hepatectomy, three who underwent MB with peroral cholangioscopy (POCS), and six in whom other devices were used. Consequently, 44 patients were included in the Conv group and 47 in the Endosheather (ES) group. Based on factors including mapping biopsy results and assessments of surgical tolerability, 18 patients in the Conv group and 15 in the ES group were judged unresectable, and 26 and 32 patients in the Conv and ES groups, respectively, underwent surgery.

This was a single-center, retrospective, observational study conducted at Shizuoka Cancer Center, where approximately 400 hepatopancreatobiliary surgeries are performed annually. This study included consecutive patients with biliary tract cancer and hilar biliary obstruction who underwent MB as part of their preoperative management from January 2017 to August 2023. The Endosheather (ES) was introduced at our institution in April 2021; patients who underwent MB before April 2021 were assigned to the Conv group, and those who underwent MB thereafter were assigned to the ES group. Patients with surgically altered anatomy, those with a history of hepatectomy, those who underwent MB with POCS, or those who underwent anything other than Conv MB or MB using ES were excluded. A flowchart of patient selection is shown in Fig. 1 . This study was approved by the Review Board of Shizuoka Cancer Center (J2023–100–2023–1-3) and was conducted in accordance with the principles of the Declaration of Helsinki. The board waived this requirement owing to the retrospective nature of the study.

### ES

**Fig. 2:**
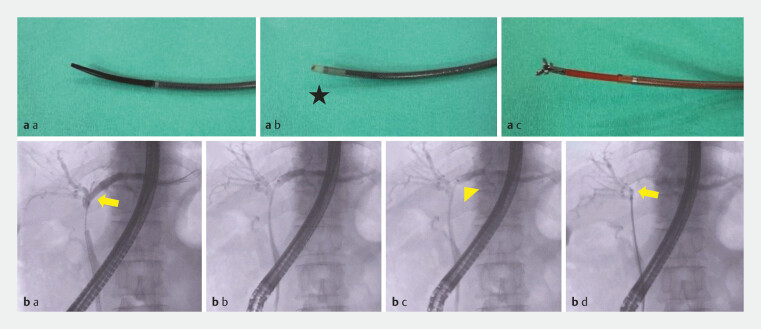
**a** Endosheather (ES) structure. a: ES is made of an inner catheter and outer sheath. The effective length is 1707 mm, and ES has two types depending on the lumen of the inner catheter (0.025 inch and 0.035 inch). The tip of the inner catheter has a tapered shape, facilitating easier insertion through the biliary stricture. b: The image of the outer sheath after removing the inner catheter. A radiopaque marker shown as a star is positioned at the tip of the outer sheath, improving visibility under fluoroscopy. c: A biopsy forceps with a diameter of 5.7F (1.9 mm), including Radial Jaw 4 (cup width, 1.6 mm), can be smoothly inserted into the outer sheath. The outer sheath has optimal rigidity and kink resistance, enabling a device to enter it repeatedly. **b** Fluoroscopic images of endoscopic retrograde cholangiopancreatography when using ES for mapping biopsy of the takeoff of the middle hepatic duct (B4) in cases when right hepatectomy was planned. a: Before ES was inserted, the takeoff of the middle hepatic duct (B4) (arrow) was confirmed with cholangiography. b: Both the inner catheter and the outer sheath were inserted along the guidewire, which was placed in the left intrahepatic duct. The ES was inserted until the radiopaque marker of the outer sheath (arrowhead) was positioned near the takeoff of B4. c: The inner catheter and guidewire were carefully removed, ensuring the position of the outer sheath remained unchanged. d: Biopsy forceps were introduced into the outer sheath to reach the takeoff of B4, and then the biopsy sample was obtained. An arrow shows the tip of the biopsy forceps.

ES consists of an inner catheter and an outer sheath ( Fig. 2 **a** ). The inner catheter is tapered at the tip to allow the guidewire to pass through it. The outer sheath is appropriately stiff, allowing it to conform to the shape of the bile duct and enabling the biopsy forceps to enter it repeatedly for sampling.

### ERCP and MB procedure

ERCP was performed under conscious sedation using a side-viewing duodenoscope (JF-260V, TJF-260V, and TJF-290; Olympus Medical Systems, Tokyo, Japan). Procedures were performed by a senior endoscopist (expert) together with fellows-in-training under direct supervision. Fellows rotated every 1 to 2 years and, therefore, did not constitute a fixed set of operators. The supervising expert was the same throughout the study period and the endoscopic drainage strategy did not change other than the introduction of ES. During ERCP, an ERCP catheter and guidewire were inserted into the bile duct, followed by sphincterotomy if there were no contraindications. A contrast agent was injected to identify the predetermined biopsy sites under cholangiography. In the Conv method, biopsy forceps are inserted directly from the duodenal major papilla into the bile duct along the guidewire and a biopsy sample is obtained under fluoroscopic guidance. When the biopsy forceps alone could not traverse the stricture, no additional devices were used. Conversely, ES was inserted over a guidewire placed in the targeted bile duct ( Fig. 2 **b** ). The inner catheter and guidewire were removed, leaving the outer sheath in place. Finally, the biopsy forceps were inserted into the outer sheath to obtain a sample from the target site under fluoroscopy. In this study, we defined the study groups (Conv and ES groups) as follows: The Conv group received MB using the Conv method, whereas the ES group received MB using ES. The biopsy forceps used for MB in the Conv group were the FB-44U-1 (Olympus Medical Systems, Tokyo, Japan), FB-45Q-1 (Olympus Medical Systems), and FB-39Q-01 (Olympus Medical Systems). Regarding the MB of the distal bile duct, the Radial Jaw 4 pediatric type (RJ4P) (Boston Scientific, Marlborough, Massachusetts, United States) was also used in some cases. In the ES group, a RJ4P was used. The details of these forceps are shown in Supplementary Fig. 1. One biopsy was performed per target site; however, if a sample could not be obtained, the procedure was repeated until the sample was visually obtained. When pathological evidence of malignancy was not confirmed before ERCP, a sample was obtained from the patient with biliary obstruction after MB. Before completing ERCP, an endoscopic nasobiliary drainage tube or a plastic stent was generally inserted into the bile duct of the future remnant lobe according to the guidelines 3 17 18 19 . To prevent post-ERCP pancreatitis, a diclofenac suppository and ulinastatin injection were administered around the time of ERCP if there were no contraindications and a pancreatic stent was inserted into the pancreatic duct, if necessary.

### Predetermined MB site

**Fig. 3:**
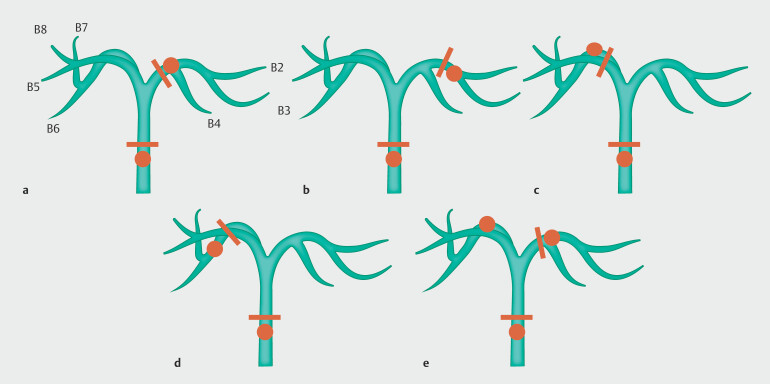
The limit of ductal transection and the biopsy sites in mapping biopsy (MB) based on the planned surgical procedure. The limit of ductal transection is shown as a black line and the MB sites are indicated by black circles. These were determined based on the planned surgical procedure from the multi-detector computed tomography images prior to endoscopic retrograde cholangiopancreatography. The limitation line on the duodenal side corresponds to the upper edge of the pancreatic head, and the MB site on the duodenal side is the distal bile duct. The MB sites were as follows. **a** Right hepatectomy: The limit of transection on the hepatic side is located at the right margin of the umbilical portion. The MB sites were at the takeoff of the middle hepatic duct (B4) and the distal bile duct. **b** Right trisectionectomy: The limit of transection on the hepatic side is located at the left margin of the umbilical portion of the portal vein. The MB sites were located where the left lateral posterior (B2) and anterior (B3) ducts branched and the distal bile duct. **c** Left hepatectomy: The transection limit on the hepatic side is located on the cranial side of the right portal vein. The MB sites were located at the casual flexure of the posterior segmental and distal bile ducts. **d** Left trisectionectomy: The limit of transection on the hepatic side is located where the posterior segmental bile duct parallels the posterior branch of the right portal vein. The MB sites are located in the peripheral portion of the posterior segmental and distal bile ducts. **e** Undetermined surgical procedure: The MB sites were at the takeoff of the middle hepatic duct (B4), casual flexure of the posterior segmental bile duct, and distal bile duct.

In radical surgery for biliary tract cancer with hilar biliary obstruction, recognizing the limits of ductal transection according to the extent of hepatectomy is crucial to ensure negative ductal margins. Based on the surgical methods determined from multi-detector CT images before ERCP, MB was performed at the site in the future remnant lobe near the resection limit, which was defined as a predetermined MB site in this study ( Fig. 3 ) 20 . Briefly, when right hepatectomy was planned, the MB sites were the takeoff of the middle hepatic duct (B4) and the distal bile duct. When left hepatectomy was scheduled, the MB sites were at the casual flexure of the posterior segmental bile duct and distal bile duct. For right trisectionectomy, the MB sites were where the left lateral posterior (B2) and anterior (B3) ducts branched along the distal bile duct. In the case of left trisectionectomy, the MB sites were the peripheral portions of the posterior segmental bile duct and the distal bile duct. Samples obtained from the distal bile duct were omitted when pancreatoduodenectomy was performed. In clinical practice, MB specimens were also obtained from sites other than the predetermined MB sites at surgeon request; however, these results were not included in this study.

### Pathological assessment of obtained specimens

Pathological information was extracted from pathological reports, which included the pathological diagnosis and quality of the specimen. Pathological diagnoses included categories such as cancer, normal, and atypical. Quality was categorized into samples with only biliary epithelium, samples with stromal tissue without epithelium, samples with both biliary epithelium and stromal tissue, and unevaluable specimens. These findings were assessed by two experienced pathologists.

### Outcome measures

Outcome measures included overall technical success rate, specimen quality, impact of MB results on the treatment strategy, and adverse events (AEs). Overall technical success was defined as cases in which the biopsy forceps reached the targeted site and samples were obtained from all predetermined MB sites. Regarding the impact of MB results on the treatment strategy, cases were evaluated in which the treatment strategy was changed based on MB results, such as transforming resectability status from resectable to unresectable cancer or changing surgical method from L2 to L3. AEs were evaluated based on the Lexicon criteria 21 . In resected cases, we calculated the negative predictive value (NPV) of MB and compared it between the Conv and ES groups. NPV was defined as the number of bile duct margins that were negative on intraoperative frozen-section pathology divided by the number of corresponding sites judged negative on MB. Information was collected from electronic medical records. These parameters were compared between the Conv and ES groups.

### Statistical analyses

Continuous variables are expressed as medians and interquartile ranges and compared using the Mann-Whitney U test. Categorical variables are expressed as percentages and compared using χ² test when all expected cell counts were ≥ 5, and Fisher’s exact test when any expected cell count was < 5 or when a cell count was zero.

We performed multivariable logistic regression analysis for technical success, including the following covariates: use of ES, Bismuth classification (I/II vs IIIa/IIIb/IV), presence of cholangitis, presence of preoperative biliary drainage, and planned extent of hepatectomy (two segments vs three segments). We also performed multivariable logistic regression analysis for adequate specimen acquisition, including the following covariates: use of RJ4P, Bismuth classification (I/II vs IIIa/IIIb/IV), presence of cholangitis, presence of preoperative biliary drainage, and planned extent of hepatectomy (two segments vs three segments). Because ES and RJ4P were always used together, they were collinear and, therefore, could not be entered simultaneously into the same model. Results are reported as odds ratios (ORs) with 95% confidence intervals (CIs); two-sided *P* < 0.05 was considered statistically significant. Analyses were conducted using R version 3.4.1 (The R Foundation for Statistical Computing, Vienna, Austria). Complete-case analysis was used; no imputation was performed.

## Results

### Patient characteristics

During the study period, 102 patients with biliary tract cancer and hilar biliary obstruction underwent MB preoperatively. Of these, 11 patients were excluded because of surgically altered anatomy (n = 1), history of hepatectomy (n = 1), MB performed with POCS (n = 3), or MB performed using other methods (n = 6). Consequently, 91 patients were evaluated (44 and 47 in the Conv and ES groups, respectively). There were no significant differences in the patient characteristics between the two groups ( Table 1 ).

**Table:** **Table 1** Patient characteristics.

	Conv N = 44	ES N = 47	P value
Male, n (%)	33 (75)	31 (66)	0.50
Age, median (IQR)	75 (47–84)	73 (45–82)	0.84
Clinical diagnosis, n			0.85
Perihilar cholangiocarcinoma	33	32	
Widely spreading cholangiocarcinoma	8	10	
Gallbladder carcinoma	1	3	
Intrahepatic cholangiocarcinoma	2	2	
Bismuth type (I/II/IIIa/IIIb/IV), n	4/8/10/8/14	2/12/14/6/13	0.69
Planned surgical procedure, n			0.39
Right trisectionectomy (plus PD)	4 (0)	1 (1)	
Left trisectionectomy (plus PD)	11 (2)	7 (0)	
Right hepatectomy (plus PD)	8 (5)	16 (5)	
Left hepatectomy (plus PD)	9 (2)	9 (4)	
Undetermined	3	4	
Pre-procedural cholangitis, yes, n (%)	8 (18)	8 (17)	0.78
Grade (mild/moderate/severe)	7/1/0	6/2/0	0.88
Pre-procedural biliary drainage, yes, n	12	20	0.19
ENBD/plastic stent	3/9	1/19	0.08
T-Bil, mg/dL, median (IQR)	2.5 (0.90–9.0)	1.5 (1.0–5.9)	0.40
CEA, ng/mL, median (IQR)	2.6 (1.9–4.6)	3.5 (2.1–5.3)	0.52
History of sphincterotomy, n (%)	12 (27)	18 (38)	1.0
Sphincterotomy on the same day as MB, n (%)	32 (73)	29 (62)	1.0
Pancreatic stent to prevent pancreatitis, n	3	3	1.0
Conv, conventional; ENBD, endoscopic nasobiliary biliary drainage; ES, Endosheather; IQR, interquartile range; MB, mapping biopsy; PD, pancreaticoduodenectomy.			

### Technical success rate

The overall technical success rate was significantly higher in the ES group than in the Conv group (59% [26/44] vs. 92% [43/47]; *P* < 0.05) ( Table 2 ). The breakdown of technical success according to MB site is shown in Table 2. Using ES improved the success rate at most MB sites; however, the success rate for the peripheral portions of the posterior segmental bile duct remained at 71%.

**Table:** **Table 2** Technical success rate of mapping biopsy.

		Conv	ES	*P* value
Overall technical success rate, % (n/N)		59 (26/44)	91.5 (43/47)	< 0.05
MB sites, % (n/N)	B2/3	75 (3/4)	100 (2/2)	
	B4	79 (11/14)	100 (17/17)	
	The c.f. of Bp	44 (7/16)	96 (26/27)	
	The peripheral Bp	69 (9/13)	71 (5/7)	
	DB	97 (34/35)	97 (35/36)	
Bp, posterior segmental bile duct; Conv, conventional; c.f., casual flexure; DB, distal bile duct; ES, Endosheather; MB, mapping biopsy.				

### Specimen quality

Table 3 shows quality of specimens, whereas Supplementary Table 1 shows MB sites and biopsy forceps used at each site. Sixty-eight and 87 specimens were evaluated in the Conv and ES groups, respectively. The proportion of samples containing both biliary epithelium and stromal tissue was significantly higher in the ES group than in the Conv group (47% [32/68] vs. 77% [67/87]; ( *P* < 0.05). The proportion of unevaluable specimens in the Conv group was significantly higher than that in the ES group (9.5% [7/68] vs. 1.1% [1/87]; *P* < 0.05).

**Table:** **Table 3** Quality of mapping biopsy specimens.

	Conv N = 68	ES N = 87	*P* value
Both biliary epithelium and stromal tissue, n (%)	32 (47)	67 (77)	< 0.05
Only biliary epithelium, n (%)	25 (38)	14 (16)	< 0.05
Stromal tissue without biliary epithelium, n (%)	4 (5.4)	5 (5.7)	1
Unevaluable specimen, n (%)	7 (9.5)	1 (1.1)	< 0.05
Conv, conventional; ES, Endosheather.			

### Impact of MB results on treatment strategy

MB results affected one (2.3%) and five (11%) patients in the Conv and ES group, respectively, although the difference was not statistically significant ( *P* = 0.11) ( Table 4 ). In the Conv group, one patient required a change in surgical method from right hepatectomy to right trisectionectomy because the sample obtained from the takeoff of B4 was cancer-positive. In the ES group, three patients needed a change in surgical procedure, whereas in two patients, the treatment strategy had to be transformed to an unresectable status. In Cases 3 and 5, MB revealed that the cancer had spread beyond the limits of ductal transection. In Case 4, cancer was detected at the takeoff of B4, making right trisectionectomy a possible surgical procedure; however, it was deemed unfeasible owing to insufficient hepatic function.

**Table:** **Table 4** Impact of mapping biopsy results on the treatment strategy.

	Cancer-positive MB site	Strategy
Conv group (n = 1)		
Case 1: Changing the surgical method	Takeoff of B4	R2→R3
ES group (n = 5)		
Case 1: Changing the surgical method	Takeoff of B4	R2→L2
Case 2: Changing the surgical method	Distal bile duct	L2→L2 plus PD
Case 3: Transforming to unresectable status	The peripheral Bp	L3→Unresectable
Case 4: Transforming to unresectable status	Takeoff of B4	R2→Unresectable
Case 5: Transforming to unresectable status	Branch of B2 and B3	R2→Unresectable
Bp, posterior segmental bile duct; Conv, conventional; ES, Endosheather; MB, mapping biopsy; PD, pancreaticoduodenectomy.		

### Adverse events

AEs are shown in Supplementary Table 2; there were no significant differences between the two groups. All the AEs were mild. Post-sphincterotomy bleeding was managed with endoscopic hemostasis, whereas other AEs were treated conservatively.

### Concordance between negative MB results and negative surgical margins

The NPV of negative MB results is shown in Supplementary Table 3. There were no significant differences in NPV between the two groups at any site. In addition, the bile duct margin findings in the three patients whose surgical procedure was modified on the basis of MB results (one in the Conv group and two in the ES group) are summarized in Supplementary Table 4.

### Factors associated with technical success

We performed univariable and multivariable analyses to identify factors associated with technical success. Use of ES was independently associated with technical success (OR 10.8, 95% CI 2.56–45.5, *P* = 0.0012; Supplementary Table 5).

### Factors associated with adequate specimen acquisition

In the overall specimen dataset, use of PRJ was an independent factor associated with adequate specimens (OR 3.18, 95% CI 1.54–6.56, *P* = 0.0017; Supplementary Table 6). In the CBD-only subset, percutaneous endoscopic jejunostomy (PRJ) use remained significantly associated with adequate specimens (OR 3.47, 95% CI 1.23–9.79, p = 0.019; Supplementary Table 7). Although the effects of ES and PRJ cannot be completely separated, these findings suggest that the acquisition of adequate specimens is largely attributable to the use of PRJ.

## Discussion

This retrospective study revealed that using ES improved the rate of technical success of MB and the quality of MB specimens in patients with biliary tract cancer, consequently affecting patient management in several patients.

The overall technical success rate was higher in the ES group than in the Conv group. In multivariable analysis of factors associated with technical success, use of ES remained independently associated with technical success. A previous study reported that the success rate of the Conv method was 64%, which is similar to that of our Conv group (62%) 22 . One reason for the improved success rate in the ES group is likely enhanced access to the intrahepatic duct beyond the biliary obstruction. When a guidewire was inserted into the targeted bile duct, both the ES and biopsy forceps were able to reach the targeted site in most cases. However, the success rate at the proximal posterior segmental bile duct did not improve even with ES. This was probably because guidewire insertion into the posterior duct was challenging and ES occasionally did not pass through the tortuous tight biliary obstruction, even along the guidewire. In some cases, a rigid guidewire can overcome these difficulties. As such, improving the success rate of proximal posterior segmental bile duct remains a challenge.

There were a few patients in whom the treatment strategy was changed based on the MB results; however, this was more frequently observed in the ES group than in the Conv group. This is probably related to improved rates of overall success and specimen quality. Larger biopsy forceps were used in the ES group than in the Conv group, which may explain why more samples containing biliary epithelium and stromal tissue were procured. This may also affect treatment strategies. In cases in which the surgical procedure was modified, two patients in the ES group had carcinoma In situ (CIS) at the hepatic ductal margin. No additional MB was performed in these patients after the change in surgical strategy, and the decision to operate was based on imaging findings. Previous reports have suggested that postoperative CIS at the ductal margin does not significantly worsen prognosis compared with R0 resection 23 , supporting appropriateness of the modified procedures. Nonetheless, to discuss the efficacy of MB, achievement of negative ductal margins should ideally be evaluated and compared between the two groups, ensuring that their backgrounds, such as pathological stage and cancer spread, are similar. We calculated and compared the NPV of bile duct margins between the two groups, but no significant differences were observed. Because the Conv group had a relatively low rate of technical success, comparisons were limited to patients who actually underwent surgery, resulting in a small sample size. Therefore, these results might have led to misleading conclusions.

POCS is another method of performing MB. The success rate of MB using POCS is 78% to 95% 14 22 . In our ES group, the success rate at the proximal posterior segmental bile duct was suboptimal and a similar trend has been observed in POCS 14 . Furthermore, one of the disadvantages of POCS is that the available forceps are small, resulting in a lower adequate tissue acquisition rate (61%) 14 . Hence, MB using ES is likely to yield outcomes similar to those achieved with POCS and cost effectiveness can be considered a strength of ES.

This study has some limitations. First, this was a retrospective study with an inherent risk of selection bias. Although this bias was mitigated to some extent by including consecutive cases, it could not be eliminated. Second, the small sample size presents a limitation because it may lead to insufficient statistical power and underestimation of the effects, such as the number of cases in which MB has an impact on the treatment plan. Third, this study was conducted at a single institution, which may restrict generalizability of the results. Differences in the protocols for preoperative evaluation and mapping of biopsy sites across institutions may influence applicability of the findings. Finally, different biopsy forceps were used for the two groups. In the Conv group, it was difficult to insert RJ4P into the bile duct; therefore, forceps smaller than RJ4P were used. In addition, because ES and RJ4P were always used together, their effects could not be separated in the multivariable analyses of factors associated with technical success and adequate specimen acquisition. Thus, it is not possible to determine with certainty whether the observed improvements in technical success and specimen adequacy are attributable to ES, RJ4P, or both. These limitations can be addressed through a prospective multicenter collaborative study.

## Conclusions

In conclusion, MB using ES for biliary duct cancers with hilar biliary obstruction improves the overall technical success rate and sample quality compared with Conv MB. This may contribute to optimization of treatment plans when assessing operability or determining surgical methods.

## Supplementary Material

Supplementary Material Zusatzmaterial
