# Supplementary material for: Utility of a novel sheath designed for mapping biopsy for preoperative malignant hilar biliary obstruction
Source: Endosc Int Open. 2026 Jan 30;14:a27871325. doi: 10.1055/a-2787-1325 (PMC12908915; doi:10.1055/a-2787-1325)

**Supplementary Table 1** Mapping biopsy sites and biopsy forceps used at each site.

| **Forceps** | **Biopsy site** | **Conv** | **ES** |
|---|---|---|---|
| FB-39Q-1 | IHB | 0 | |
| | DB | 4 | |
| FB-45Q-1 | IHB | 6 | |
| | DB | 24 | |
| FB-44U-1 | IHB | 23 | |
| | DB | 1 | |
| Radial Jaw 4 | IHB | 0 | 44 |
| | DB | 8 | 35 |

Conv, conventional; DB, distal bile duct; ES, Endosheather; IHB, intrahepatic bile duct.

Endosc Int Open | DOI 10.1055/a-2787-1325 | © The Author(s).

**Supplementary Table 2** Adverse events.

| | Conv<br>N = 44 | ES<br>N = 47 | *P* value |
|---|---|---|---|
| All, n (%) | 3 (6.8) | 4 (9.6) | 1 |
| Pancreatitis, n (%) | 3 (6.8) | 1 (2.4) | 0.35 |
| Cholangitis, n (%) | 0 (0) | 2 (4.8) | 0.50 |
| Bleeding, n (%) | 0 (0) | 1 (2.4) | 1 |

Conv, conventional; ES, Endosheather.

**Supplementary Table 3** Negative predictive value of mapping biopsy.

| | | | Conv | ES | *P* value |
|---|---|---|---|---|---|
| MB sites | B2/3 | The number of negative in MB, n | 0 | 1 | |
| | | NPV, (%) | 0/0 (0) | 0/1 (0) | 1 |
| | B4 | The number of negative in MB, n | 5 | 12 | |
| | | NPV, (%) | 4/5 (80) | 9/12 (75) | 0.67 |
| | The c.f. of Bp | The number of negative in MB, n | 2 | 8 | |
| | | NPV, (%) | 2/2 (100) | 5/8 (63) | 0.58 |
| | The peripheral Bp | The number of negative in MB, n | 3 | 3 | |
| | | NPV, (%) | 3/3 (100) | 3/3 (100) | 1 |
| | DB | The number of negative in MB, n | 17 | 16 | |
| | | NPV, (%) | 14/17 (82) | 13/16 (81) | 1 |

Bp, posterior segmental bile duct; c.f., casual flexure; Conv, conventional; DB, distal bile duct; ES, Endosheather; MB, mapping biopsy; NPV, negative predictive value.

**Supplementary Table 4** Postoperative ductal margin status in cases with modified surgical methods.

| | Cancer-positive MB site | Strategy | Ductal margin |
|---|---|---|---|
| Conv group | | | |
| Case 1: Changing the surgical method | Takeoff of B4 | R2→R3 | Negative ductal margin |
| ES group | | | |
| Case 1: Changing the surgical method | Takeoff of B4 | R2→L2 | Carcinoma in situ In hepatic ductal margin |
| Case 2: Changing the surgical method | Distal bile duct | L2→ L2 plus PD | Carcinoma in situ In hepatic ductal margin |

Bp, posterior segmental bile duct; Conv, conventional; ES, Endosheather; MB, mapping biopsy; PD, pancreaticoduodenectomy.

**Supplementary Table 5** Factors associated with technical success.

| Factor | Univariate analysis | | | Multivariate analysis | | |
|---|---|---|---|---|---|---|
| | Odds ratio | 95% CI | *P* value | Odds ratio | 95% CI | *P* value |
| Sex, male | 1.34 | 0.47-3.83 | 0.583 | | | |
| Bismuth type, I or II | 11.10 | 1.41-87-70 | 0.02240 | 7.74 | 0.84-71.2 | 0.071 |
| Planned surgical procedure, R2 or L2 | 9.67 | 3.22-29.00 | 0.000053 | 5.87 | 1.65-20.80 | 0.006 |
| Planned surgical procedure, R2 or R3 | 3.02 | 0.998-9.15 | 0.0505 | | | |
| Pre-procedural biliary drainage, yes | 1.11 | 0.396-3.11 | 0.841 | | | |
| Pre-procedural cholangitis, yes | 0.792 | 0.224-2.81 | 0.718 | | | |
| History of sphincterotomy, yes | 3.00 | 0.971-9.26 | 0.0562 | | | |
| Use of ES | 10.20 | 2.73-37.80 | 0.00055 | 10.80 | 2.56-45-50 | 0.001 |

CI, confidence interval; ES, Endosheather; L2, Left hepatectomy; R2, Right hepatectomy; R3, Right trisectionectomy.

**Supplementary Table 6** Factors associated with adequate tissue.

| Factor | Univariate analysis | | | Multivariate analysis | | |
|---|---|---|---|---|---|---|
| | **Odds ratio** | **95% CI** | ***P* value** | **Odds radio** | **95% CI** | ***P* value** |
| Sex, male | 0.64 | 0.29-1.39 | 0.258 | | | |
| The use of RJ4P | 3.53 | 1.75-7.13 | 0.000428 | 3.43 | 1.69-6.96 | 0.000664 |
| Bismuth type, I or II | 0.90 | 0.44-1.84 | 0.772 | 0.924 | 0.434-1.97 | 0.838 |
| Planned surgical procedure, two-section hepatectomy | 1.27 | 0.59-2.73 | 0.546 | | | |
| Pre-procedural cholangitis, yes | 0.43 | 0.17-1.13 | 0.086 | 0.471 | 0.173-1.28 | 0.140 |
| Specimen from intrahepatic duct | 1.13 | 0.58-2.21 | 0.718 | 1.10 | 0.542-2.23 | 0.795 |
| Pre-procedural biliary drainage, yes | 1.71 | 0.83-3.54 | 0.146 | | | |
| No history of sphincterotomy | 1.06 | 0.44-2.52 | 0.903 | | | |

CI, confidence interval; RJ4P, Radialjaw4 pediatric type.

**Supplementary Table 7** Factors associated with adequate tissue from distal bile duct.

| Factor | Univariate analysis | | | Multivariate analysis | | |
|---|---|---|---|---|---|---|
| | **Odds ratio** | **95% CI** | ***P* value** | **Odds ratio** | **95% CI** | ***P* value** |
| Sex, male | 0.466 | 0.147-1.48 | 0.195 | | | |
| The use of RJ4P | 4.12 | 1.50-11.30 | 0.00588 | 3.550 | 1.260-10.00 | 0.0163 |
| Bismuth type, I or II | 1.40 | 0.483-4.06 | 0.536 | | | |
| Planned surgical procedure, two-section hepatectomy | 1.02 | 0.368-2.80 | 0.977 | | | |
| Pre-procedural cholangitis, yes | 0.726 | 0.199-2.65 | 0.628 | | | |
| Pre-procedural biliary drainage, yes | 3.50 | 1.12-10.90 | 0.0309 | 2.830 | 0.866-9.24 | 0.0851 |
| No history of sphincterotomy | 0.845 | 0.251-2.84 | 0.786 | | | |

RJ4P, Radial Jaw 4 pediatric type.

**Supplementary Fig. S1** Biopsy forceps used in this study. (a) FB-39Q-1 (Olympus Medical Systems, Tokyo, Japan): cup width, 1.8 mm; cup length, 3.2 mm; and opening width, 4.2 mm. (b) FB-45Q-1 (Olympus Medical Systems): unilateral-opening forceps: cup width, 1.8 mm; cup width, 3.2 mm; and opening width, 3 mm. (c) FB-44U-1 (Olympus Medical Systems): cup width, 1.1 mm; cup length, 2.8 mm; and opening width, 4.1 mm. (d) Radial Jaw 4 pediatric type (Boston Scientific, Marlborough, MA, USA): cup width, 1.6 mm; cup length, 2.8 mm; and opening width, 5.4 mm.

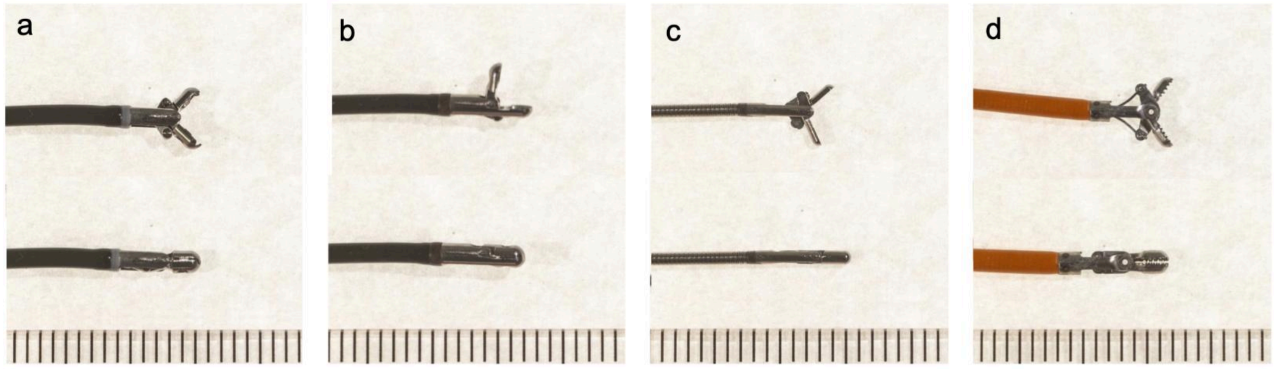

Supplement: Supplementary file 1 — Supplementary Material [file 10-1055-a-2787-1325_27889403.pdf]
